# Interaction between Plate Make and Protein in Protein Crystallisation Screening

**DOI:** 10.1371/journal.pone.0007851

**Published:** 2009-11-16

**Authors:** Gordon J. King, Kai-En Chen, Gautier Robin, Jade K. Forwood, Begoña Heras, Anil S. Thakur, Bostjan Kobe, Simon P. Blomberg, Jennifer L. Martin

**Affiliations:** 1 Division of Chemistry and Structural Biology, Institute for Molecular Bioscience, University of Queensland, Brisbane, Queensland, Australia; 2 School of Chemistry and Molecular Biosciences, University of Queensland, Brisbane, Queensland, Australia; 3 ARC Special Research Centre for Functional and Applied Genomics, University of Queensland, Brisbane, Queensland, Australia; 4 School of Biological Sciences, University of Queensland, Brisbane, Queensland, Australia; Weizmann Institute of Science, Israel

## Abstract

**Background:**

Protein crystallisation screening involves the parallel testing of large numbers of candidate conditions with the aim of identifying conditions suitable as a starting point for the production of diffraction quality crystals. Generally, condition screening is performed in 96-well plates. While previous studies have examined the effects of protein construct, protein purity, or crystallisation condition ingredients on protein crystallisation, few have examined the effect of the crystallisation plate.

**Methodology/Principal Findings:**

We performed a statistically rigorous examination of protein crystallisation, and evaluated interactions between crystallisation success and plate row/column, different plates of same make, different plate makes and different proteins. From our analysis of protein crystallisation, we found a significant interaction between plate make and the specific protein being crystallised.

**Conclusions/Significance:**

Protein crystal structure determination is the principal method for determining protein structure but is limited by the need to produce crystals of the protein under study. Many important proteins are difficult to crystallise, so that identification of factors that assist crystallisation could open up the structure determination of these more challenging targets. Our findings suggest that protein crystallisation success may be improved by matching a protein with its optimal plate make.

## Introduction

Protein crystallography is the major structural biology technique, accounting for more than 80% of solved protein structures, and commercially available 96-well plates are essential components of modern high-throughput protein crystallisation condition screening [1]. Vapour diffusion is the most frequently used technique for protein crystallisation [2], [3] and is exploited in two common formats, hanging drop and sitting drop. Both formats involve setting two solutions, the crystallisation condition or reservoir solution and the protein drop, within a sealed plate well. The protein drop contains the protein mixed with the crystallisation condition. The hanging and sitting drop vapour diffusion formats utilise 96-well plates in different ways. For the hanging drop method, the protein drop is suspended above the well solution from a seal and makes no contact with the crystallisation plate. For the sitting drop method, the protein drop sits directly on a surface of the crystallisation plate above the crystallisation condition.

Protein crystallisation is a complex phenomenon involving the following three processes (1) the slow concentration of components within the protein drop during equilibration so that the protein becomes supersaturated, (2) crystal nucleation, and (3) crystal growth. For crystallisation condition screening, the first two of these three processes are the most important as the aim is to identify conditions that can be optimised for crystal growth rather than to produce diffraction quality crystals.

Many studies have examined the effect of the crystallisation condition ingredients or the plate geometry on the rate of equilibration of the protein drop with the crystallisation condition and both can markedly affect the quality of the crystals [4], [5]. Recently, a new model for the equilibration processes was reported for the hanging drop crystallisation format [6]. This model extends previously proposed models designed to describe the rate of equilibration of hanging drop crystallisation experiments, and the authors consider the effect of well geometry, drop to reservoir distance, drop volume and precipitant type on the rate of equilibration. The nucleation process has also been studied in detail. Attempts to control nucleation have been made including seeding with protein micro-crystals [7], or introducing heterologous nucleating agents, such as mineral surfaces [8], porous material with pore sizes on the order of magnitude of protein molecules [9], and diverse agents such as dried seaweed, horsehair, cellulose and hydroxyapatite [10], [11]. Other workers have shown that nucleation is affected by the nature of the surface on which the protein drop is set [12]. In general, these studies indicate that for some proteins, particular types of surfaces can promote nucleation and crystallisation, though no universal nucleation promoter has yet been identified. Furthermore, no systematic study has been reported of the effect on protein crystallisation of different commercial plate makes. We therefore set out to investigate the effect of plate make on protein crystallisation success.

## Results

We first performed a pilot study to investigate 10 commercially available plate makes designed for sitting-drop vapour diffusion protein crystallisation, and a commercially available hanging-drop plate format used routinely in our laboratories (**Table 1**). We used six test proteins, varying in mass from 15 to 60 kDa, that crystallise under different conditions (*eg* salt or PEG precipitants, pH range 4.0–8.5). We assessed the crystallisation success of the plates as the number of drops that gave rise to crystals for each of the 6 proteins. The 11 plates tested appeared to fall into three different classes. Plates 1, 4, 8, 9 and 11 group together in the class with the highest crystallisation success (56–58 out of a maximum possible 72 drops with crystals); plates 2, 3 and 7 are in the middle class (49–51 drops with crystals) and plates 5, 6 and 10 are in the lowest scoring class (41–44 drops with crystals) (**Table 1**). The water permeability of each plate was also investigated over the 37-day experimental time-frame, to determine if the observed crystallisation success may be related to dehydration. However, only small differences in dehydration were measured (**Table 1**) and these did not correlate with the observed crystallisation success of the plates. These initial results did suggest that different plate make might give rise to different protein crystallisation success but the conclusions are limited because just one of each plate make was included in the study.

**Table 1:** Crystallisation plates used in this study.

Plate	1	2	3	4	5	6	7	8	9	10	11^a^
**Material** b	Polystyrene	Polystyrene	Polystyrene	Polypropylene	COC	COC	COC	PZero	Polystyrene	Patented polymer	Polystyrene (plate) polycarbonate (tape)
**Sitting-drop well Volume** (µL)	2	3.9	4.3	3	10	7	4	2	10	5	—
**Distance** c **(mm)**	7.6	3.5	10.5	7	9.1	9.1	8.8	8.9	8.8	8.4	11
**Number of drops with crystals (max 72)**	57	49	51	58	41	42	50	56	57	44	58
**PEG conc** g **% (w/v)**	11.73±1.12#	10.91±0.53#	10.51±0.06#	10.29±0.06#	10.00±0.06	10.00±0.06	10.00±0.06	10.73±0.24#	10.51±0.06#	10.00±0.06	10.90±0.06#
**Water absorption** h	<0.4%	<0.4%	<0.4%	0.03%	0.01%	0.01%	0.01%	—	<0.4%	—	Plate <0.4% Tape 0.1%

a This plate was set up for hanging-drop crystallisation, using an adhesive hanging-drop tape rather than a drop well for the protein drops; information on both the plate and the tape is given.

b Plate material is taken from manufacturer's information. COC; cyclic olefin copolymer, PZero; Zero polarization polymer.

c Distance from the bottom of the reservoir to the bottom of the sitting-drop well or to the hanging-drop tape, as per manufacturer's information.

d N_tot_ is the total number of drops with crystals at 37 days, with a theoretical maximum value of N_tot_ is 72.

g PEG 4000 concentration (% w/v) after 37 days is averaged for 12 replicates (see methods). Values are given for mean (±95% confidence intervals, or where no variation was observed in the 12 replicates, error calculated from the error of the instrument).

# The PEG 4000 concentration after 37 days is significantly different from the starting concentration of 10% (w/v).

h Values for water absorption (after immersion at 23°C for 24 hours) of the plate material are taken from the Goodfellow index of materials (www.goodfellow.com; Goodfellow Corporation, Oakdale, PA, USA) (and for COC from www.polyplastics.com/en/product/lines/topas/TOPAS.pdf Polyplastics Co., Ltd., Tokyo, Japan). We were unable to find values for plate material of plates 8 and 10 but our PEG 4000 concentration results suggest that the former is water permeable and the latter has very low water permeability.

We therefore extended our study to allow a more statistically rigorous analysis. We chose three proteins and three plate makes for further study: two of the plate makes were sitting drop plates representative of the highest and lowest scoring classes from the pilot study (Plates 1 and 5, respectively) and the third plate make was the hanging drop plate (Plate 11) that we use routinely and which was also classified in the highest scoring class in the pilot study. We designed an experiment in which nine copies of each of the three plate makes were tested in crystallisation experiments.

We first examined the resulting data for evidence of variations in crystallisation success as a consequence of position on the 96-well plate, which might for example indicate that edge wells behave differently to wells in the central region of the plate. We found there was no effect of column number or row number on crystallisation fraction (rows: β = –0.010, SE = 0.062, z = –0.166, p = 0.868; columns: β = –0.015, SE = 0.014, z = –1.040, p = 0.299) (see Materials and Methods for further description of these parameters). We therefore excluded row and column number from further analyses.

We then used the data set to test the hypothesis that plate make has a significant effect on crystallisation success, as our pilot data had suggested. However, the model that allowed different “within-plate make” variances for the three plate makes was not a significantly better fit to the data than the simpler, equal variance model (LR χ^2^_5_ = 1.683, p = 0.891). We could not, therefore, detect significant differences in the variability in crystallisation fraction across the three plate makes, for the proteins and conditions used. Estimates of the standard deviations in crystallisation fraction for each plate make were, Plate 11: 0.205, Plate 1: 0.347, Plate 5: 0.346. The equal variance model produced a standard deviation of 0.347, 95% HPD interval: (0.339, 0.582). Overall, none of the plate makes was more variable than any other suggesting that plates are manufactured very consistently.

**Figure 1:**
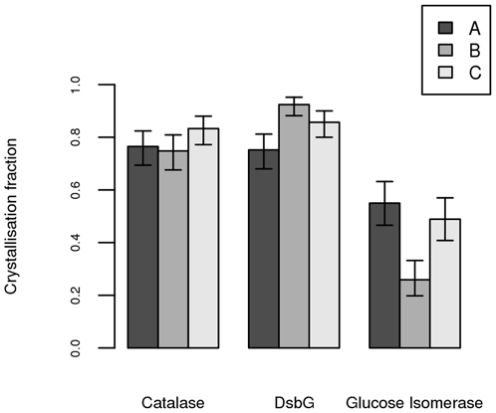
Mean crystallisation fraction for the three proteins on three different plate makes. A is plate 11, B is plate 1 and C is plate 5. Error bars are 95% confidence intervals. Data were collected from twenty seven 96-well plates (nine plates of each make) and a total of 864 wells for each protein over the three plate makes.

Finally, we examined the data for an interaction between plate make and protein on crystallisation success rate. We found that there was a statistically significant interaction between plate make and protein (p<10^−6^), indicating that plate makes differ in their ability to grow crystals with different proteins. The analysis presented in **Figure 1** shows that catalase has consistently high crystallisation fractions across all three plate makes, while for DsbG plate 11 is statistically inferior to plate 1, but not to plate 5. The third protein, glucose isomerase, showed the lowest crystallisation fractions for all plates, with plate 1 being particularly unsuitable for crystallisation of this protein, under the conditions used in this experiment.

## Discussion

Our findings indicate that crystallisation success may be improved by matching a protein with its optimal plate make. In protein crystallisation condition screening using vapour diffusion, the type of plate used could potentially contribute to three effects on crystallisation success: an effect on the intrinsic rate of equilibration of the sealed experiment, an effect on the equilibration end-point due to changes in the crystallisation conditions through dehydration, or an effect on the nucleation of protein crystals. Plate geometry is known to have an effect on the rate of equilibration of the protein drop [13]. Plate-associated dehydration could well contribute to increased crystallisation success, though our pilot study suggested that any dehydration effect was small and slow and did not correlate with crystallisation success. The third possible plate effect is a contact effect, where the surface of the plate may provide, for some proteins, a more or less effective template for crystal nucleation. There are many reported examples of heterogeneous nucleating agents on protein crystallisation [13], [14] and a specific surface interaction that leads to either a promotion or an inhibition of crystallisation for some proteins is perhaps the most likely explanation for the observed interaction between plate make and protein.

In conclusion, the major finding of this work is that quality control of protein crystallisation requires optimisation of conditions, including the plate make, and this has never been addressed before. The correct way to optimise conditions is the use of properly designed experiments and appropriate statistical analyses [15], [16], though such studies are rare in the literature. While the present knowledge does not allow one to predict the best match of a protein to a plate make, we speculate that these issues may be important for industrial production of protein crystals in large quantities, and for the study of proteins that are difficult or slow to crystallise.

## Materials and Methods

### Pilot Study of Plate Effect on Protein Crystallisation

#### Crystallisation plates

Ten commercially available 96-well sitting-drop crystallisation plates (**Table 1**) were selected on the basis of diversity of material and sitting-drop well geometry. In the hanging-drop setup, the drops were prepared on a hanging-drop seal (TTP4150-5100 sourced from Millennium Science, Victoria, Australia) that was compatible with the Mosquito® nanolitre crystallisation robot (TTP LabTech, Melbourn, UK).

#### Proteins

Six proteins were used: hen egg-white lysozyme (Roche Diagnostics Corporation, Indianapolis, USA), bovine liver catalase and *Thaumatococcus daniellii* thaumatin (Sigma-Aldrich, Missouri, USA), *Streptomyces rubiginosus* glucose isomerase (Hampton Research, California, USA), *Eschericheria coli* DsbG [17] and mouse latexin [18]. The crystallisation conditions for each have been reported previously, though it is not known whether these are optimised conditions. As necessary, the proteins were dissolved or dialysed and concentrated for crystallisation using 10 kDa cut-off centrifugal concentrators (Millipore, Massachusetts, USA) in the buffers listed in **Table 2**. Protein concentration was measured using the Bradford assay (Bio-Rad™, California, USA).

**Table 2:** Proteins and crystallisation conditions used in this study.

Protein	Lysozyme^1^	Catalase^1^	Latexin	Glucose isomerase^2^	DsbG^3^	Thaumatin^1^
**Molecular Weight (kDa)**	14.7 (Monomer)	57.6 (Monomer)	25.7 (Monomer)	43.2 (Monomer)	51.4 (Dimer)	22 (Monomer)
**Protein concentration used/reported (mg/ml)**	54/75	10/10	11/7	24/20	13/13	40/50
**Protein buffer**	100 mM Na acetate, pH 4.8, 0.02% (w/v) Na azide	25 mM Tris-HCl, pH 7.0	25 mM Tris-HCl, pH 7.0	6 mM Tris-HCl, pH 7.0, 1 mM MgSO_4_	25 mM HEPES, pH 7.0, 50 mM NaCl	MilliQ Water
**Crystallisation condition**	25 mM Na acetate, pH 4.8, 0.02% (w/v) Na azide, 1.1 M NaCl^4^	100 mM Tris-HCl, pH 8.5, 8% (w/v) PEG 8000^5^	100 mM cacodylate, pH 6.5, 1.8 M (NH_4_)_2_SO_4_ 6	100 mM HEPES, pH 7.2, 1.4 M (NH_4_)_2_SO_4_ 4	100 mM Na citrate, pH 4.0, 22% (w/v) PEG 4000, 200 mM (NH_4_)_2_SO_4_ 7	0.1 M ADA, pH 6.5, 1 M K/Na tartrate^8^

1 Lysozyme, catalase and thaumatin were dissolved in their respective protein buffers.

2 Glucose isomerase was dialysed for 24 hours at 4°C in its protein buffer.

3 DsbG was exchanged into its protein buffer prior to concentration.

4 From Rigaku Corporation crystallisation procedures (http://www.rigaku.com).

5 Hampton Research crystal screen I condition 36 (http://www.hamptonresearch.com). ADA, N-(2-acetamido)-iminodiacetic acid; HEPES, 4-(2-hydroxyethyl) piperazine-1-ethanesulfonic acid; Tris, tris(hydroxymethyl)aminomethane.

6 See reference [18].

7 See reference [17].

8 See reference [23].

#### Crystallisation

Three crystallisation conditions were used for each protein: the precipitant at the published crystallisation concentration, the precipitant at a concentration 20% lower than the published condition and the precipitant at a concentration 20% higher than the reported precipitant concentration (buffer and other additives conditions remained the same in these three test conditions). Four replicates were used for each of these three conditions. All the crystallisation experiments were set up on the same day, in parallel using the same protein batches (incubated on ice) for each plate. Protein buffers and crystallisation conditions for each protein are given in **Table 2**. Plates were removed from their plastic sleeves just prior to setting up the experiments to minimise contamination. The crystallisation condition (85 µl) was placed in the reservoirs of the crystallisation plates using a Biomek® 2000 Laboratory Automation workstation (Beckman Coulter, California, USA). The protein drops constituted 200 nL of protein and 200 nL of crystallisation solution and were prepared using a Mosquito® robot (TTP LabTech, Melbourn, UK) at room temperature. All sitting-drop plates were sealed using tape (Qiagen, California, USA) and all plates were incubated in the same incubator (Thermoline, Queensland, Australia) set to 20°C. In total, 792 crystallisation drops were prepared in one day. Each of the 11 plates used in this pilot study held 4 replicates of 3 crystallisation conditions for 6 proteins. Images of the crystallisation experiments were captured using a Crystal Monitor™ workstation (Emerald Biosystems, Washington, USA) at the standard settings of 1.0 brightness, 1.0 gamma adjustment and auto exposure for the highest image resolution (10 s per image). The brightness was adjusted to 1.25 or 1.5 for plates with dark shadowing around the crystallisation drop to improve the image quality.

#### Analysis

To evaluate crystallisation success, images of crystallisation drops were taken on days 1, 2, 3, 6, 9, 16, 23, 30 and 37. For each of these days and for each plate, the number of protein drops with crystals was recorded. Crystallisation success, N_tot_, for each plate was defined as the total number of protein drops with crystals on day 37. Four replicate wells of the three conditions were set up for each of the six proteins in each of the 11 plates; therefore the theoretical maximum and minimum N_tot_ values for each plate were 72 and 0, respectively.

#### Crystallisation condition dehydration

The refractive index of PEG solutions was measured using a Bausch and Lomb Abbe 60 refractometer (Bellingham & Stanley, London, UK) and used to calculate PEG concentration as described previously [4]. Twelve 85 µL replicates of a 10% w/v PEG 4000 solution were pipetted into the same 10 types of sitting drop plates and one hanging drop crystallisation plate used above, and sealed and incubated as described above. The refractive index of each reservoir solution was measured before and after a 37 day incubation period and the final PEG 4000 concentration determined using a plot of refractive index versus PEG 4000 concentration (7% to 20% (w/v)) calculated using Prism version 4 (GraphPad Software, California, USA). The mean and 95% confidence intervals of the PEG 4000 reservoir concentrations were calculated using Excel (Microsoft, Washington, USA).

### Comprehensive Study of Plate Effect on Protein Crystallisation

The experiments for this follow-up statistically rigorous study were designed to increase the power of the data by increasing sample size and by decreasing the number of variables by using constant conditions wherever possible. We therefore chose not to use different batches of protein, or different batches of crystallisation solutions, but instead produced sufficient quantities of each protein and of the crystallisation solutions to perform all 27 plate experiments on the same day under the same conditions. This approach controls the variability that would result from batch to batch variation of the protein or the crystallisation condition. We chose proteins that are known to crystallise, and we chose conditions under which these proteins are known to crystallise.

#### Crystallisation plates

Two sitting drop plate makes were chosen based on their performance in the preliminary analysis, one from the low success group, Plate 5, and one from the high success group, Plate 1. We also included the hanging drop plate, Plate 11, because it is routinely used in our labs and scored well in the pilot study.

#### Proteins

Three proteins were used for the comprehensive study: bovine liver catalase, *Streptomyces rubiginosus* glucose isomerase and *Eschericheria coli* DsbG and prepared as described for the initial study (**Table 2**).

#### Crystallisation

Both hanging- and sitting-drop experiments were set up using the Mosquito® nanolitre crystallisation robot (TTP LabTech, Melbourn, UK). Each drop was built by adding 200 nL of the crystallisation condition to 200 nL of the protein solution. The crystallisation conditions are listed in **Table 2**.

A total of 27 plates, nine of each of the three plate makes, were set with crystallisation drops. Each protein was represented on every one of the 27 plates by either 24 or 36 drops, and a total of 864 wells were set for each protein (total of 2,592 wells altogether). Plates were incubated at 20°C in a Formulatrix RockImager (Formulatrix, Massachusetts USA). The crystallisation drops were imaged with a Formulatrix RockImager at: 0, 1, 2, 3, 5, 8, 13 and 21 days. The presence of crystals in a well at 21 days was taken as a crystallisation success.

#### Statistical analysis

We used binomial generalised linear mixed-effects models (GLMMs) with a logit link to analyse the crystallisation fractions (fraction of wells containing crystals). All analyses were performed in R 2.7.1, using the lme4 package [19], [20]. We treated plate make (3 levels, one for each plate make) and protein (3 levels: catalase, DsbG, and glucose isomerase) as fixed factors, and row number and column number for each well as covariates. Within-make, plate-to-plate variation was accounted for by including plate ID as a random factor (Equation 2).
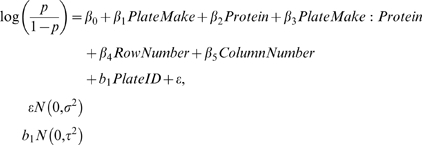
(2)


The experimental design allowed us to test whether crystallisation fraction varied with plate make or protein, with row or column number, and to quantify individual variation within and among plates. Only wells where the crystallisation drop had been set successfully were included in the analysis: 64 wells were removed owing to poor set-up of the drop by the robot. Drops were scored positive for crystallisation if crystals appeared on or before day 21. Tests of fixed effects are problematic in mixed-effects models because the distribution of the fixed effects is uncertain under the null hypothesis. In particular, denominator degrees of freedom for F tests are difficult to determine (Bates, personal communication). To circumvent this problem, we tested the significance of the protein type–plate make interaction by sampling from the posterior distribution of the fixed effects using Markov Chain Monte Carlo (e.g. [21]). We took 10^6^ samples from the joint posterior distribution of the fixed effects, checked the traces for lack of convergence, and examined histograms of the marginal posterior distributions. We obtained a p-value for this interaction under the null hypothesis of a mean of zero, against the alternative of a general elliptic multivariate distribution. This allowed us to test whether the effect of plate make on the crystallisation fraction varied according to protein. We tested whether the within-make variation differed due to plate make, to examine whether any plate make had more consistent results than the others. To test this hypothesis, we fitted a model that allowed different variances for the plate makes, and compared it to a model with equal variance for all plate makes, using a likelihood ratio test. We examined quantile-quantile plots of the residuals and the random effects to look for departures from normality, and plots of the residuals versus the fitted values to check for heteroscedasticity and nonlinearity in the residuals [22].
